# Aminolyzed Polycaprolactone Nanofiber Scaffolds with Visible Light-Activated Sterilization for Tissue Engineering Applications

**DOI:** 10.1021/acs.biomac.5c01352

**Published:** 2025-10-07

**Authors:** Robert Willimetz, Pavel Kubát, Jan Svoboda, Jana Musílková, Jiří Mosinger

**Affiliations:** † Faculty of Sciences, 112302 Charles University, Hlavova 2030, Prague 2,, 128 43, Czech Republic; ‡ J. Heyrovský Institute of Physical Chemistry, v.v.i., Academy of Sciences of the Czech Republic, Dolejškova 3, Prague 8,, 182 23, Czech Republic; § 86879 Institute of Macromolecular Chemistry, Academy of Sciences of the Czech Republic, Heyrovského nám. 2, Prague 6,, 162 00, Czech Republic; ∥ Institute of Physiology of the Czech Academy of Sciences, Vídeňská 1083, Prague 4,, 142 00, Czech Republic

## Abstract

New photoactive nanofiber materials based on an aminolyzed polycaprolactone membrane with demonstrated cytocompatibility were developed. Two photoactive compounds, the photosensitizer Rose Bengal and the nitric oxide photodonor 4-nitro-3-(trifluoromethyl)­aniline, were covalently bonded to the nanofiber surface, with or without a glutaraldehyde linker. The surface functionalization was confirmed via X-ray photoelectron spectroscopy, UV–vis absorption, and steady-state and time-resolved luminescence spectroscopy. Upon excitation with green or blue light, these materials efficiently generate antibacterial species, including singlet oxygen, with a slight contribution of hydrogen peroxide and nitric oxide. A potent light-induced antibacterial effect was demonstrated against *Escherichia coli*. Furthermore, the functionalized photoactive membranes, especially those with a glutaraldehyde linker and photosterilized by light, not only excluded the material toxicity but also demonstrated improved cell adhesion and proliferation when tested with adipose tissue-derived stem cells. These materials, which offer a unique combination of light-controlled surface sterilization and high cellular compatibility, are promising for advanced tissue engineering applications.

## Graphical abstract

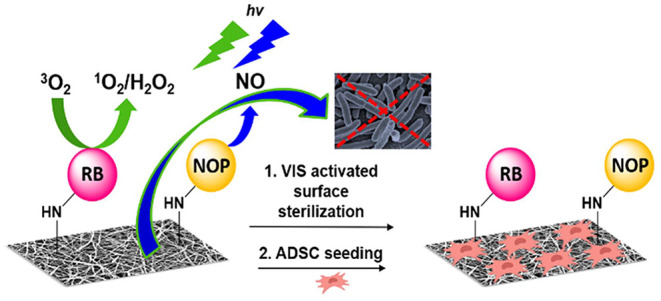

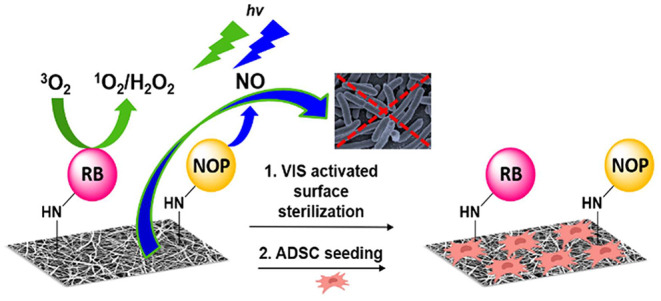

## 1 Introduction

The rapid development of novel nanomaterials based on polymer nanofibers for a broad range of applications, such as scaffolds for tissue engineering,^1,2^ wound dressings^3^ and filtration materials,^4^ has received much attention. These materials with nanofiber diameters of approximately 100–400 nm are characterized by a high surface area, transparency to light, high oxygen permeability/diffusion, and a nanoporous structure,^5,6^ which prevent bacteria and other pathogens from passing through the nanofiber materials because they are retained on the surface.^7^

Tissue engineering is a rapidly growing interdisciplinary field in which nanofiber materials could have many important applications as bioactive scaffolds that mimic the architecture of tissue.^8–10^ However, the specific properties of the material, such as its hydrophilicity, surface microstructure, chemical composition, and roughness,^11^ strongly influence the cell–material interaction at the interfacial region; therefore, these parameters must be considered. This, together with an effective and safe method of sterilization of nanofibers, represents one of the greatest challenges for developing new suitable materials for tissue engineering applications.^12^

The porous structure of the scaffold surface used for tissue replacements typically leads to improved cell adhesion and proliferation. However, this porous structure raises concerns about increased bacterial colonization and it is necessary to find effective strategies to prevent colonization, especially by Gram-negative bacteria.

To date, many biodegradable and biocompatible synthetic polymers, such as polycaprolactone (PCL) or polylactic acid (PLA), have been used in a broad range of biomedical applications, such as drug delivery systems and regenerative medicine.^13,14^ However, one of the major drawbacks of the utilization of these synthetic polymers is poor hydrophilicity and therefore poor cell attachment, which can lead to inefficiency of the scaffold in creating an interface with good contact/adherence with living cells.^15,16^

In recent years, aminolysis has been frequently used as a facile and straightforward wet chemical method to introduce reactive primary amino onto the surface of scaffolds via the reaction of diamine compounds with polyesters while preserving the bulk physicochemical properties.^15,17^ This method led to favorable changes in surface wettability^15,18^ and improved cell proliferation^17^ than did pristine polyesters, without considerable changes in mechanical properties.^19^ A substantial improvement in aminolyzed surfaces toward more rigid surfaces with enhanced cell adhesivity is the utilization of glutaraldehyde as a cross-linking agent.^20^

The crucial step before the application of nanofiber materials as scaffolds for tissue engineering applications is their sterilization.^12^ Generally, several common methods of sterilization of nanofiber scaffolds are employed, such as heat treatment, gamma or UV irradiation, and plasma or chemical sterilization.^12,21,22^ These techniques often have undesirable side effects that can lead to not only mechanical or morphological changes^23–25^ but also changes in the toxicity of the scaffold due to harmful residues from the sterilization process.^12^

Recently, we developed nanofiber materials with encapsulated porphyrinoid photosensitizers that generate O_2_(^1^Δ_g_) with a high quantum yield upon irradiation with visible light.^26,27^ The small diameter of the nanofibers allows the efficient diffusion of O_2_(^1^Δ_g_) outside the nanofibers and the photooxidation of chemical or biological targets. The short-lived, highly cytotoxic O_2_(^1^Δ_g_) efficiently kills bacteria such as *Escherichia coli*, *Staphylococcus aureus*, and *Pseudomonas aeruginosa*,^28–31^ nonenveloped polyomaviruses, and enveloped baculoviruses^32^ only on the surfaces of such nanofiber materials. Recently, these nanofiber materials have been successfully applied in dermatology as antibacterial wound coverings activated by visible light.^31^ A common feature of such polymeric nanofiber materials with encapsulated photoactive photosensitizers is a low diffusion length for O_2_(^1^Δ_g_) (typically tens to hundreds of nm),^33^ which limits the efficiency of the photooxidation of chemical/biological targets close to the nanofiber surface; therefore, it can be a suitable method for surface sterilization.

Alternatively, the photogeneration of NO radicals from NO photodonors encapsulated in nanofiber materials can also be applied for sterilization, as demonstrated in our previous studies.^26,34^ NO radicals have a lifetime of approximately 4 s in air and a diffusion radius of approximately 100 μm. Like O_2_(^1^Δ_g_), NO is characterized by its small size, absence of charge, multitarget therapeutic capability with a broad spectrum of antibacterial activity, and absence of multidrug resistance problems that are encountered with several conventional target-specific drugs.^35^ The combination of O_2_(^1^Δ_g_) with NO represents an interesting strategy given the bimodal antibacterial treatments/surface sterilization. The objective of this study was to prepare functionalized PCL nanofiber materials that can bind a suitable photosensitizer to ensure surface sterilization and NO photodonor to ensure sterilization in the entire volume of the membrane and to exploit the possible additive antibacterial effect due to the photogeneration of two antibacterial agents upon visible light activation. The high surface area and nanoporous structure of nanofiber materials are advantages that provide a high concentration of photoactive compounds and a highly specific surface for tissue engineering. To the best of our knowledge, the visible light sterilization of nanofiber membranes for tissue engineering via the photogeneration of O_2_(^1^Δ_g_) and NO has not been studied to date.

In this study, we focused on the aminolysis of electrospun polycaprolactone (PCL) nanofiber membranes and their functionalization with/without a glutaraldehyde linker for the covalent bonding of the Rose Bengal (RB) photosensitizer and/or 4-chloro-2-(trifluoromethyl)-1-nitrobenzene NO photodonor (NOP). Rose Bengal was selected as common photosensitizer with a broad absorption bands in the green and yellow region of the visible spectrum generating O_2_(^1^Δ_g_) with high quantum yield (Φ_Δ_ ∼ 0.75),^36^ NOP with absorption in blue region was selected for his efficient photogeneration of NO.^37,38^

The photooxidative, photoantibacterial/photosterilization properties, as well as the in vitro cellular response of adipose-derived stems were investigated for potential use of such membranes in tissue engineering.

## 2 Experimental Section

### 2.1 Chemicals

Polycaprolactone (PCL, *M*_n_ = 80 000), 1,3-diaminopropane (≥99%), Rose Bengal sodium salt (dye content 95%), 4-nitro-3-(trifluoromethyl)­aniline (98%), glutaraldehyde solution (50 wt % in H_2_O), isopropanol (G. R., ISO reagent), acetic acid (≥99%), LB agar, LB medium, KI and other inorganic salts were purchased from Sigma–Aldrich and used as received.

### 2.2 Electrospinning

PCL was dissolved in a mixture of DCM:DMF (3:2 (v:v)) to prepare a 10 wt % solution for electrospinning. The solution was stirred for 18 h at room temperature and subsequently loaded in a 20 mL syringe with a 21 G stainless steel needle. The fibers were collected on a grounded aluminum foil collector at a distance of 11 cm from the needle tip. The PCL nanofiber membrane (1, Figure 1) was electrospun for 30 min with an applied voltage of 11 kV and a flow rate of 0.7 mL/h.

**Figure 1:**
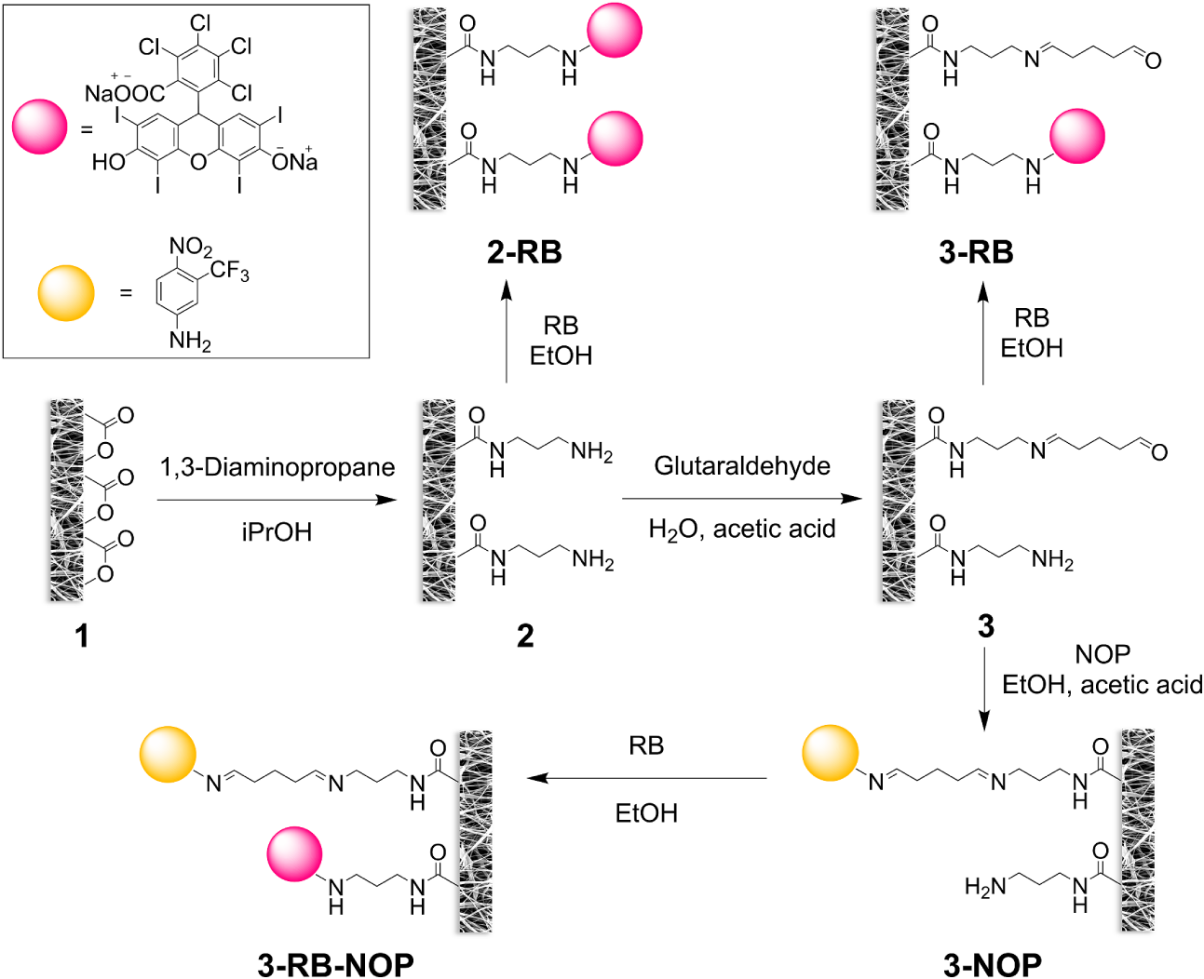
Scheme illustrating postprocessing of the electrospun PCL membrane and binding of RB (pink) and/or NOP (yellow).

### 2.3 Aminolysis of PCL Electrospun Membranes

A sample (28 cm^2^) of the pristine PCL nanofiber membrane (1) was immersed in 40 mL of 10 wt % 1,3-diaminopropane solution in isopropanol in a sealed glass vial. The vial was placed in a shaking incubator, and the aminolysis process was carried out at 37 °C for 72 h with shaking at 150 rpm. The aminolyzed PCL membrane (2) was subsequently washed with pure isopropanol and deionized water and then stirred for 2 h in 300 mL of an EtOH: deionized water mixture (1:4 (v:v)) to ensure that all the 1,3-diaminopropane was completely washed away. Finally, the membrane was dried in a vacuum desiccator overnight and stored at 5 °C for further use.

### 2.4 Determination of Primary Amino Groups on the Membranes

The presence of primary amino groups on the surface of 2 or 3 was quantitatively determined via the Orange II dye method.^39^ A piece (2.5 cm^2^) of membrane 2 or 3 was immersed in 2.0 mL of Orange II dye in acidic water (pH 2.0, adjusted with 1 M HCl). The reaction proceeded for 30 min at 40 °C. After the reaction, the resulting membranes were washed several times with acidic solution, stirred in large amounts of acidic solution for 30 min to completely remove unbound dye, and then air-dried at 37 °C. The desorption of Orange II dye from the membranes was performed in 2.9 mL of alkaline solution (pH 12, adjusted with NaOH) for 30 min at 40 °C. After desorption, the pH of the resulting solutions was adjusted to 2.0 by the addition of 50 μL of 6 M HCl. The desorbed Orange II dye was monitored via UV–vis spectroscopy at 484 nm. The amount of chemically bound Orange II was estimated from the calibration curve via UV–vis detection at 484 nm (Figure S4). The pristine PCL membrane (1), as a control, was treated under the same conditions.

### 2.5 Binding of Glutaraldehyde on the Membranes

A sample of membrane 2 (28 cm^2^) was prewetted in EtOH and stirred in 20 mL of 5 wt % glutaraldehyde solution (50% glutaraldehyde in water) with 50 μL of acetic acid for 6 h at 40 °C. After the reaction, the resulting membrane 3 was washed three times with deionized water and stirred in a deionized water:EtOH solution (1:1 (v:v)) for 6 h. The amount of chemically bound glutaraldehyde was estimated indirectly as the diminishing amount of free amino groups on 3 compared with 2 using the Orange II method. The presence of covalently bonded glutaraldehyde on the surface of 3 was also detected qualitatively via Schiff’s test, which is based on colorimetric detection of free aldehyde groups. Membrane 3 was incubated with 4 mL of freshly prepared Schiff’s reagent. After 10 min, the sample developed a deep magenta color, indicating the presence of aldehyde functional groups. The sample was then washed with excess deionized water and air-dried in the dark. The color of each sample was monitored via UV–vis spectroscopy. The pristine PCL membrane (1) was subjected to the same procedure as a control.

### 2.6 Binding of NOP on Membranes

Membrane 3 (28 cm^2^) was immersed in 20 mL of NOP solution in EtOH (1 mg/mL) with a catalytic amount of acetic acid. The reaction proceeded with shaking at 200 rpm for 16 h at 40 °C in the dark. The resulting 3-NOP membrane was washed with deionized water and EtOH and further stirred in a deionized water:EtOH solution (1:1 (v:v)) for 6 h.

### 2.7 Binding of RB on the Membranes

A sample of membrane 2, 3, or 3-NOP (28 cm^2^) was immersed in a 15 mL solution of RB in EtOH (1 mg/mL) and stirred in the dark for 2 h at 200 rpm. After the reaction, the resulting membranes with covalently bonded RB (2-RB, 3-RB, or 3-RB-NOP) were thoroughly rinsed multiple times with deionized water and EtOH and subsequently stirred in 500 mL of an EtOH: deionized water mixture (2:1 (v:v)) for 24 h in the dark. The washing solution was changed several times. Finally, the membrane was dried in a vacuum desiccator overnight and stored at 5 °C for further use.

### 2.8 Scanning Electron Microscopy (SEM)

The nanofiber morphology was studied using a scanning electron Quanta 200 FEG microscope (FEI, Czech Republic).

### 2.9 Apparent Contact Angle Measurements

The hydrophobic nature of the PCL surfaces was characterized by performing apparent contact angle (ACA) measurements using a surface energy evaluation system (See System, Czech Republic).^27^

### 2.10 X-ray Photoelectron Spectroscopy (XPS)

All XPS measurements were performed using a K-Alpha^+^ XPS spectrometer (Thermo Fisher Scientific, UK), which operates at a base pressure of 1.0 × 10^–7^ Pa. The samples were analyzed using a microfocused, monochromated Al Kα X-ray source at an angle of incidence of 30° (measured from the surface) and an emission angle normal to the surface. The kinetic energy of the electrons was measured using a 180° hemispherical energy analyzer operated in constant analyzer energy mode (CAE) at 200 and 50 eV pass energies for the survey and high-resolution spectra, respectively. To limit the X-ray-induced destruction of the samples and maximize the signal-to-noise ratio, 204 individual points were measured over the sample surface. At each point, high-resolution core levels and survey spectra were measured. Spectral resolutions of 1.0 and 0.1 eV were used for the survey and high-resolution spectra, respectively. Data acquisition and processing were performed with Thermo Avantage software. All measured spectra were charged with reference to the C 1s contribution at a binding energy of 285.0 eV attributed to the C–C and C–H moieties.

### 2.11 UV–Vis Absorption and Fluorescence Spectroscopy

The UV–vis absorption spectra were recorded using Unicam 340 and Cary 4000 spectrometers. The steady-state fluorescence spectra were monitored using an FLS 980 spectrofluorimeter (Edinburgh Instruments, UK).

### 2.12 RB Release Experiments

RB release experiments with 2-RB, 3-RB, and 3-RB-NOP membranes were conducted to evaluate the stability of the bound dye under near-physiological conditions, including temperature, pH, and ionic strength. A membrane sample (28 cm^2^) was left in a 0.1 M phosphate-buffered saline (PBS) solution (pH = 7.4, *I* = 0.15 M) in the dark for 24 h at 37 °C with mild stirring at 60 rpm. The absorption spectra were subsequently measured, and the values at 561 nm were monitored.

### 2.13 Time-Resolved Near-Infrared Luminescence of O_2_(^1^Δ_g_)

The time-resolved near-infrared phosphorescence of O_2_(^1^Δ_g_) at 1270 nm was monitored using a Ge detector (Judson J16-8SP-R05M-HS) with excitation by a Lambda Physik COMPEX 102 excimer laser (λ_exc._ = 308 nm).^33^

### 2.14 Photo-Oxidation of Iodide by Nanofiber Membranes

A piece (2 cm^2^) of the 2-RB, 3-RB, or 3-RB-NOP membrane fixed on quartz glass was placed in a quartz cell (4 cm^3^) that contained iodide detection solution.^40^ The cell was irradiated with a visible light 18 W Rubylux green LED (λ_exc._ = 515 nm, see Figure S12) from a 27 cm distance. The changes in the UV–vis absorbance at 351 nm, which were attributed to the formation of I_3_^–^, were recorded and compared with those of a blank solution of the same composition that was stored in the dark or an irradiated solution in the presence of D_2_O or NaN_3_.

### 2.15 Detection of Hydrogen Peroxide

For detection of hydrogen peroxide, a scopoletin assay was used. In this assay, fluorescent scopoletin was oxidized by photoproduced H_2_O_2_ under catalysis by horseradish peroxidase (HRP) to a nonfluorescent form.^41^

### 2.16 Detection of Nitric Oxide (NO)

Chemical detection of photoreleased NO from membrane 3-NOP and 3-RB-NOP was performed using the Griess assay. The experimental details are described in our previous study (see also Supporting Information).^26^

### 2.17 Antibacterial Tests

Antibacterial assays were performed on *Escherichia coli* DH5α (Invitrogen, California, USA) with the plasmid pGEM11Z (Promega, Wisconsin, USA) bacterial strain. The experimental details are described in the Supporting Information.

### 2.18 Cytocompatibility Tests

Pristine samples (1) and aminolyzed samples (2) (controls) aminolyzed samples with RB bonded via glutaraldehyde (3-RB) and with a combination of RB and NOP (3-RB-NOP)) were selected for cytocompatibility tests. Before the cells were seeded, the tested materials were inserted into polystyrene cultivation plates (TPP, Trasadingen, Switzerland). The samples were exposed to a green LED light (λ_exc_ = 515 nm) for 15 min at a distance of 20 cm from both sides. In some cases, the samples (3-RB and 3-RB-NOP) were subsequently irradiated by a blue LED light (λ_exc._ = 414 nm, see Figure S12) for 15 min to observe the effect of photogeneration of NO (from 3-RB-NOP) against the control (3-RB). ADSC (human adipose-derived stem cells) were seeded 5 min after irradiation at a seeding density of 30,000 cells/cm^2^, i.e., approximately 18,000 cells/well. The cells were cultured for 8 days in DMEM (Sigma–Aldrich, MO, U.S.A., Cat. No. M4892) supplemented with 10% fetal bovine serum (FBS; Sebak GmbH, Aidenbach, Germany) and gentamicin (40 μg/mL, LEK, Ljubljana, Slovenia) at 37 °C in an air atmosphere with 5% CO_2_. The behavior of the cells on the samples was evaluated after 3 and 8 days of cultivation for each type of sample and for each time interval.

### 2.19 Evaluation of Cell Number

On days 3 and 8 after cell seeding, the cells on the samples were rinsed with PBS and fixed with 4% paraformaldehyde for 15 min. The cell nuclei were visualized via fluorescence DNA staining using Hoechst #33258 (Sigma–Aldrich, MO, U.S.A.) at a concentration of 5 μg/mL PBS for 1 h. The cell nuclei, which emit blue fluorescence, were photographed with an Olympus IX71 epifluorescence microscope (10× objective) equipped with a DP 80 digital camera (both from Olympus, Japan) and counted on microphotographs. The number of cells was counted partially automatically with Fiji ImageJ software, a scientific image analysis tool (National Institutes of Health and the Laboratory for Optical and Computational Instrumentation, University of Wisconsin). In addition, the cell number was replenished and verified by a precise manual counting of the cells on the microphotographs because the samples were 3D and the software was not able to distinguish all the cell nuclei in the image with different degrees of focus of cells that colonized scaffold in deeper layers.

Statistical analysis: The cell numbers were calculated as the arithmetic mean ± standard deviation (S.D.) from 40 to 60 microphotographs obtained in total by counting cells from three independent samples for each experimental group and time interval. The statistical analysis was carried out using SigmaStat (Jandel Corporation, USA), one-way analysis of variance (ANOVA), and the Student–Newman–Keuls method. A value of *p* ≤ 0.05 was considered significant.

### 2.20 Cell Morphology

On day 8 after seeding, the cell morphology was evaluated. Filamentous actin (F-actin) was visualized by staining with phalloidin conjugated with Alexa Fluor 488 (green fluorescence, Cat. No. A12379) to evaluate the assembly of the actin cytoskeleton and the shape and spreading of cells on the tested materials. The samples were stained for 1 h at room temperature, rinsed with PBS and photographed under a Leica Stellaris 8 confocal microscope (Leica Microsystems, Mannheim, Germany; 40× objective, oil immersion). This approach can suppress the autofluorescence of the scaffold material and refine proper cell visualization. Consequently, three independent samples of each modified scaffold type were evaluated and documented in 5 microphotographs for each.

## 3 Results and Discussion

### 3.1 Preparation and Basic Characterization of Nanofiber Membranes

The preparation of pristine electrospun PCL membranes via electrospinning (1) as well as all postprocessing modifications resulting in membranes being aminolyzed (2), aminolyzed with bonded RB (2-RB), aminolyzed with bonded glutaraldehyde (3), and bonded with NOP (3-NOP) or RB (3-RB) or both (3-RB-NOP) via glutaraldehyde are described in the Experimental Section and schematically depicted in Figure 1.

Figure 2 shows the morphology of the nanofiber membranes (2-RB) and histograms of the nanofiber diameters for all the membranes with average diameters and apparent contact angles. SEM micrographs of other nanofiber membranes are depicted in Figure S1. All the data show that surface modification by aminolysis of the pristine electrospun PCL membrane and after the binding of the glutaraldehyde, RB, or NOP photoactive compounds on the surface does not considerably affect the morphology of the nanofiber structure, which has a diameter between 150 and 400 nm.

**Figure 2:**
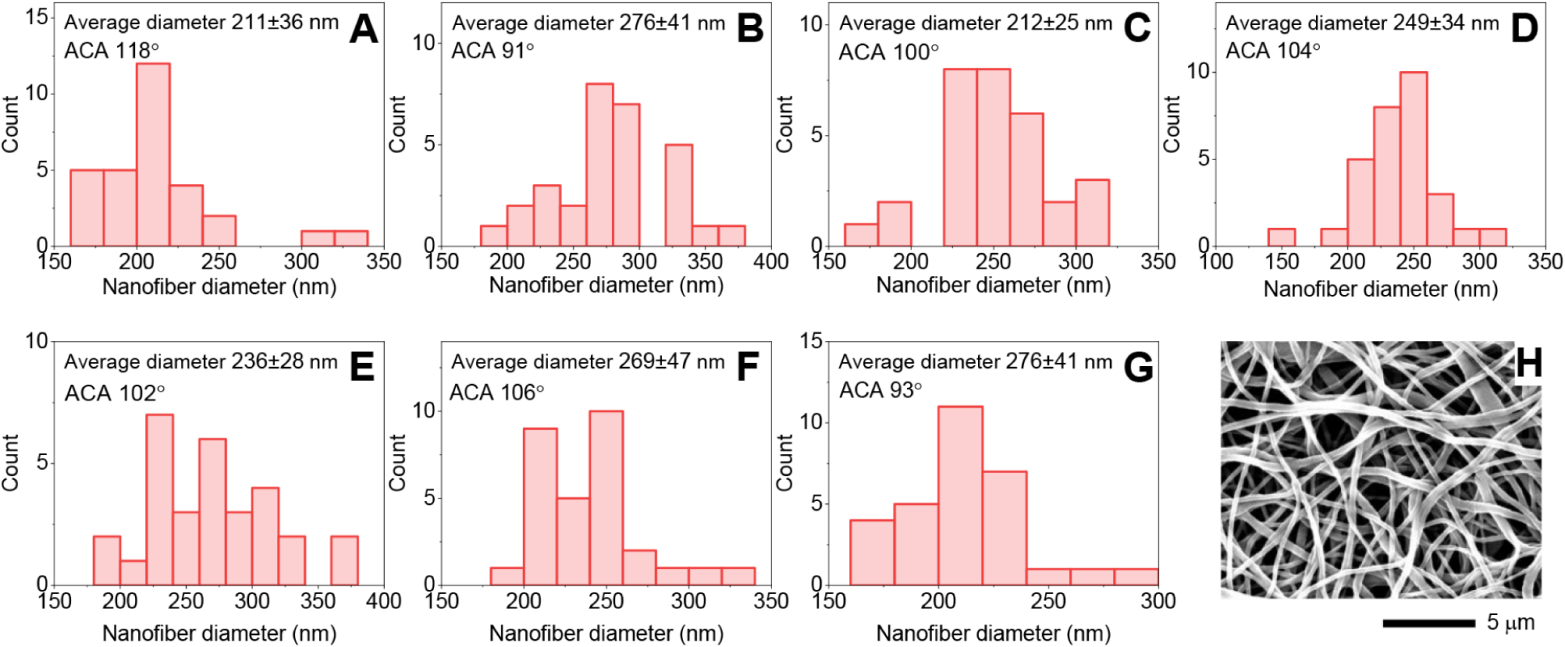
Histograms of nanofiber diameters for membranes 1 (A), 2 (B), 2-RB (C), 3 (D), 3-NOP (E), 3-RB (F), and 3-RB-NOP (G) with an average diameter of nanofibers and apparent contact angles and a representative SEM micrograph of the 3-RB-NOP membrane (H).

After postprocessing, the membranes did not exhibit any significant morphological changes, even under harsh aminolysis conditions. The membranes also exhibited minor irregularities, such as nodes and bundles of nanofibers, typical of nanofiber membranes prepared via electrospinning.^42^

Another important parameter, especially for the application of photoactive nanofiber materials that release short-lived species such as O_2_(^1^Δ_g_), is their hydrophilic/hydrophobic character. The electrospun pristine PCL nanofiber membrane (1) exhibited hydrophobic properties with an apparent contact angle (ACA) of approximately 118°. The slight effect of aminolysis on surface wettability was confirmed, as sample 2 had ACA values of approximately 91° (Figure S2). The enhanced hydrophilicity was partially diminished after surface functionalization by RB and/or NOP. This effect was expected due to the decreasing number of free polar amine groups on the surface. Nevertheless, all the functionalized membranes exhibited slightly increased hydrophilicity. Notably, the ACA method, which is commonly used for differentiating between the hydrophobic and hydrophilic natures of nanofiber samples, provides only qualitative information and can be used only for comparisons of samples with the same or similar structures.

### 3.2 Postmodification Proofs

To confirm the surface modification of the pristine PCL membrane (1), sufficiently sensitive methods are needed for the detection of primary amino and aldehyde groups. For colorimetric detection of primary amine groups, the Orange II dye method was used. The method was previously validated for quantifying free primary amine groups on aminolyzed PET films.^3,7^ The results (Figure S3) indicate successful aminolysis of PCL by 1,3-diaminopropane, yielding a surface concentration of amino groups on the sample 2 of (1.2 ± 0.2) × 10^–11^ mol/cm^2^. Subsequent modification of the sample 2 with glutaraldehyde was indirectly confirmed by a marked decrease in both adsorption and desorption of the Orange II dye on and off the sample 3, which exhibited a significantly lower surface amine concentration of (2.5 ± 0.4) × 10^–12^ mol/cm^2^. This corresponds to an approximately 4.8 ± 1.1-fold reduction in primary amine content, consistent with effective glutaraldehyde conjugation.

The presence of glutaraldehyde on membrane 3 was qualitatively detected via Schiff’s reagent (see Experimental Section). The strong purple coloration of the sample containing free −CHO groups was observed and detected via UV–vis spectroscopy, in contrast to that of control 1 (Figure S5).

### 3.3 X-ray Photoelectron Spectroscopy

The chemical compositions of the pristine and modified PCL membranes were confirmed via XPS, and the atomic percentages are summarized in Table 1.

**Table 1:** Chemical Composition of the Membranes Determined by XPS in Atomic %

	C 1s C–C	C 1s C–O	C 1s C=O	N 1s	O 1s	I 3d_5/2_	Cl 2p_3/2_	F 1s
1	52.54	12.70	12.10		22.65	-	-	-
2	51.13	12.70	12.07	0.19	23.91	-	-	-
2-RB	51.08	12.90	12.47	0.19	23.24	0.05	0.06	-
3	50.74	12.88	11.90	0.18	24.31	-	-	-
3-RB	51.72	13.01	11.44	0.24	23.50	-	-	0.09
3-NOP	51.24	12.91	11.95	0.27	23.52	0.05	0.06	-
3-RB-NOP	51.42	13.12	11.88	0.31	23.07	0.06	0.08	0.05

To limit the X-ray-induced destruction of samples and maximize the signal-to-noise ratio, 204 individual points were measured over the sample surface, and the resulting spectra were obtained as averages. The high-resolution C 1s spectra could be fitted into the peaks corresponding to C–C carbons at 285.0 eV, C–O carbons at 286.5 eV and C=O carbons at 288.9 eV originating from PCL. Successful aminolysis was confirmed by the presence of a nitrogen N 1s signal at 399.8 eV, corresponding to uncharged nitrogen (Figure 3A). The successful binding of RB in samples 2-RB, 3-RB, and 3-RB-NOP was confirmed by the presence of a spin-split Cl 2p_3/2_ → Cl 2p_1/2_ doublet (main contribution centered at 200.8 eV, separation between contributions of 1.7 eV, Figure 3B) and I 3d_5/2_ → I 3d_3/2_ doublet (main contribution centered at 620.3 eV, separation between contributions of 11.6 eV, Figure 3C), clearly indicating the presence of the organic chlorine Cl–C and iodine I–C moieties. The binding of NOP in samples 3-NOP and 3-RB-NOP was confirmed by the presence of an F 1s singlet at 697.8 eV corresponding to the −CF_3_ group (Figure 3D).

**Figure 3:**
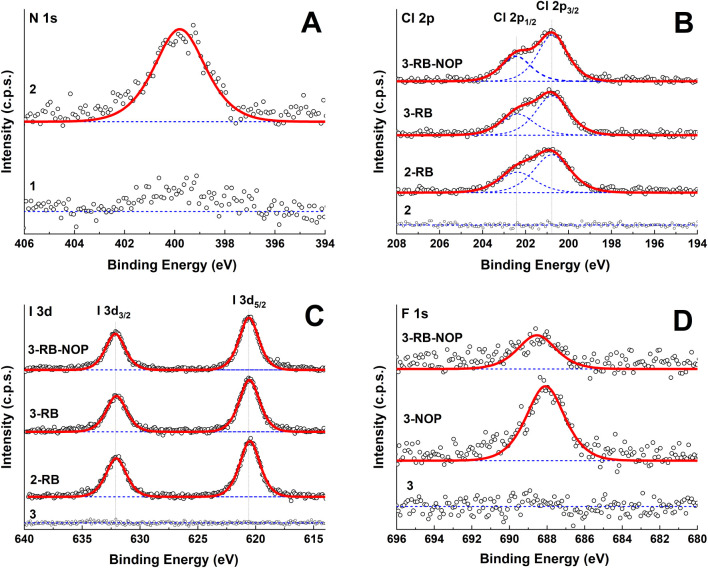
High-resolution N 1s (A), Cl 2p (B), I 3d (C), and F 1s (D) XPS spectra of PCL nanofiber membranes. The measured XPS spectra are presented with open circles, whereas their corresponding fitted envelopes are presented with red lines. The individual contributions are represented with blue lines.

### 3.4 Spectral Analysis

UV–vis absorption and fluorescence spectra were measured to evaluate the effects of the postprocessing modifications and to control the presence of the bonded photoactive compounds. For example, the UV–vis absorption spectra of RB in EtOH (Figure 4A) and 2-RB (Figure 4B) confirmed the presence of bound RB on the membrane, with a typical maximum at 559 nm and no marked aggregation or shift in the RB absorption maximum upon binding. Additionally, the fluorescence spectra were similar, with a slight redshift of the fluorescence band from 573 nm (EtOH) to 585 nm (2-RB) due to a less polar environment (Figure 4C). Additionally, the fluorescence spectra of 3-RB and 3-RB-NOP demonstrated successful binding of RB on the membranes.

**Figure 4:**
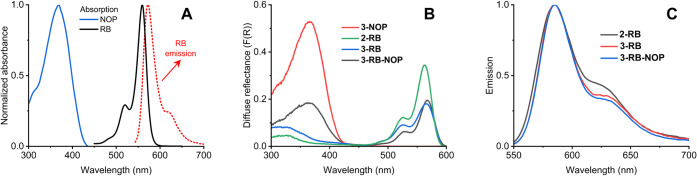
Normalized UV–vis absorption and fluorescence spectra of RB (black and red) and NOP (blue) in EtOH (A), diffuse reflectance (B), and fluorescence (C) spectra of 2-RB (black), 3-NOP (blue), 3-RB (red), and 3-RB-NOP (green).

Similarly, the UV–vis absorption spectra of NOP in EtOH (Figure 4A) and 3-NOP (Figure 4B) were similar. No NOP fluorescence was observed in any of the tested samples.

### 3.5 Kinetics of Singlet Oxygen O_2_(^1^Δ_g_)

To quantify the photogeneration of O_2_(^1^Δ_g_), direct measurements of O_2_(^1^Δ_g_) luminescence at 1270 nm were performed for all the membranes with RB photosensitizers (2-RB, 3-RB, and 3-RB-NOP). The kinetic profiles of the O_2_(^1^Δ_g_) luminescence were fitted to a single-exponential decay function for calculation of the O_2_(^1^Δ_g_) lifetime (τ_Δ_). The calculated values of τ_Δ_ are similar for all three membranes (*ca*. 11–13 μs, Figure 5) and are higher than those previously reported for photogeneration with a chlorine e6 photosensitizer encapsulated in PCL nanofibers (∼8 μs).^26^ This difference corresponds to the different environments of RB molecules covalently bonded to nanofiber surfaces in comparison with previously published data for photosensitizers encapsulated in the interior of nanofibers.^26,43^

**Figure 5:**
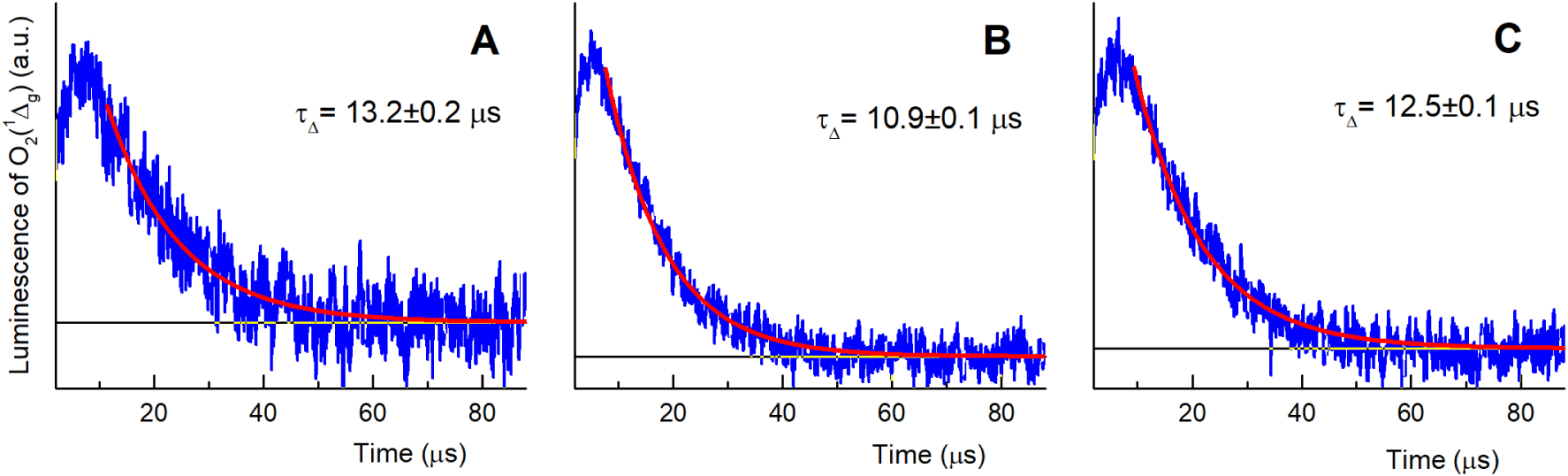
Time-resolved luminescence of O_2_(^1^Δ_g_) at 1270 nm observed for membranes 2-RB (A), 3-RB (B), and 3-RB-NOP (C), calculated as the difference between luminescence in an oxygen atmosphere and vacuum. The red lines represent single exponential fits to the experimental data.

### 3.6 Photooxidation of External Substrates

The ability of the samples to photo-oxidize external substrates was tested via a sensitive iodide method. The produced concentration of I_3_^–^ is proportional to the concentration of photogenerated O_2_(^1^Δ_g_).^40^ The samples of photoactive membranes (2-RB, 3-RB, and 3-RB-NOP) were irradiated directly in iodide detection solution. The kinetics of photooxidation increased in D_2_O, as the lifetime of O_2_(^1^Δ_g_) was approximately 18 times greater than that in H_2_O.^44^ In contrast, the kinetics were nearly zero in the presence of NaN_3_, an effective physical quencher of O_2_(^1^Δ_g_).^40^ No photooxidation was observed in the dark (Figure 6A). No leakage of RB or NOP from the nanofiber membrane to the detection solution was detected (Figure S6).

**Figure 6:**
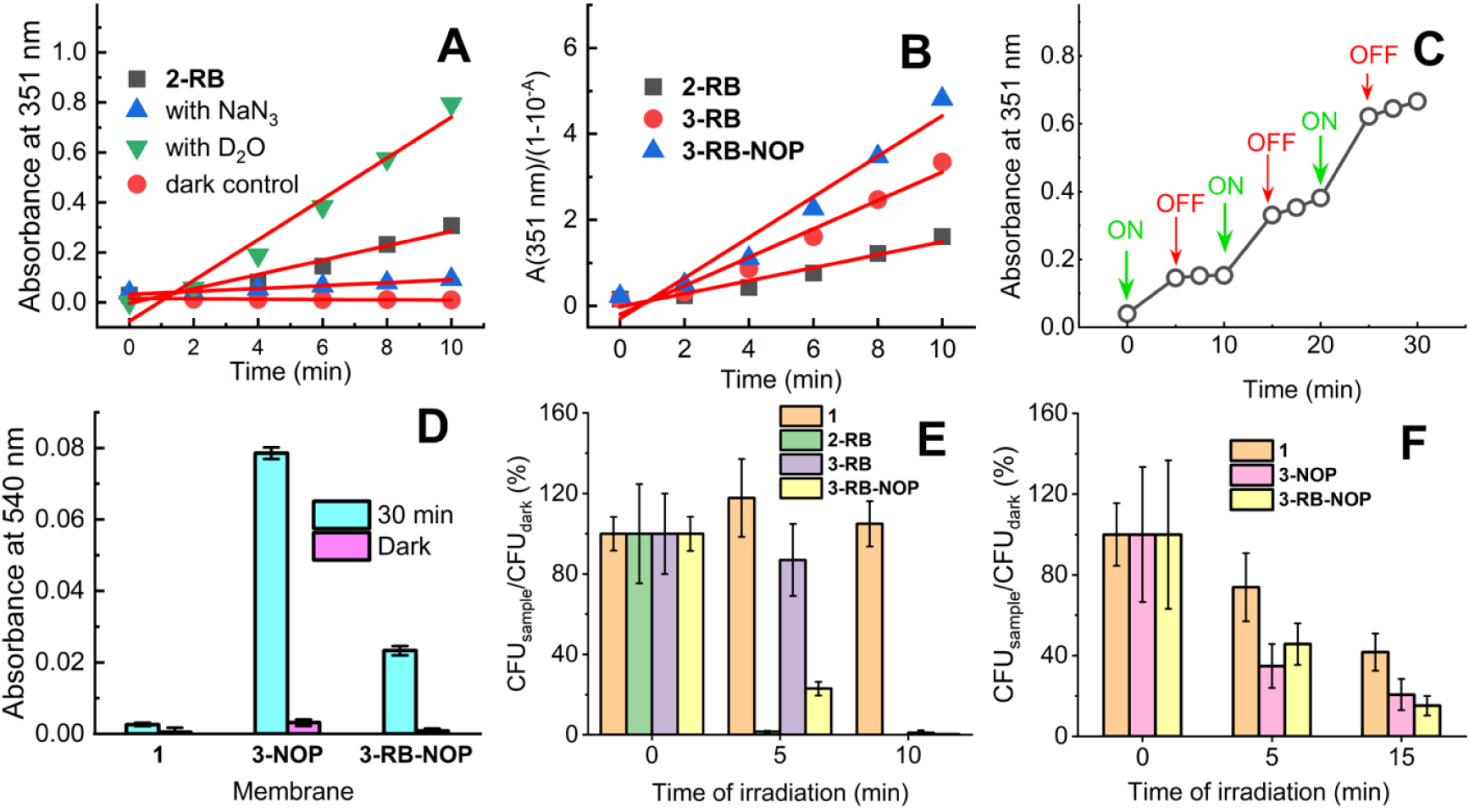
Detection of O_2_(^1^Δ_g_): (A) Kinetics of I_3_^–^ absorbance at 351 nm for 2-RB in iodide detection solution, D_2_O, 0.02 M NaN_3_, and the dark control. (B) The relative photooxidation efficacy of 2-RB, 3-RB, and 3-RB-NOP estimated as kinetics of I_3_^–^ absorbance related to the absorption at excitation light in a detection solution containing a piece of membrane. (C) Demonstration of the ON/OFF character of the photooxidation of the iodide detection solution using sample 3-RB-NOP; “ON” and “OFF” correspond with the start and end of irradiation, respectively. (D) NO was detected via a Griess assay. Changes in the absorbance at 540 nm for 3-NOP and 3-RB-NOP indicate the formation of purple azo dyes (λ_exc._ = 414 nm). (E) Antibacterial test: CFU of *E. coli* on agar plates after inoculation with bacteria harvested from the surfaces of 1, 2-RB, 3-RB, and 3-RB-NOP stored in the dark (0 min) and after 5 and 10 min of green light irradiation and incubation overnight (λ_exc._ = 515 nm). (F) Antibacterial test: CFU of *E. coli* on agar plates after inoculation with bacteria harvested from the surfaces of 1, 2-RB, 3-RB, and 3-RB-NOP stored in the dark (0 min) and after 5 and 15 min of blue light irradiation and incubation overnight (λ_exc._ = 414 nm).

Typically, the differences in the photooxidation kinetics of the tested samples depend on the intensity of the light absorbed. Therefore, the relative photooxidation efficacies (PEs) of 2-RB, 3-RB, and 3-RB-NOP were compared and calculated as the slope of the dependence of A­(I_3_^–^)/(1–10^–A^) on irradiation time, where A­(I_3_^–^) is the absorbance of the photoproduced I_3_^–^ at 351 nm, and A is the absorption of a sample at the excitation wavelength (λ_exc._ = 515 nm). For the rough photooxidation ability, see Figure S7. The photooxidative activity of 3-RB-NOP was further evaluated by irradiating the sample with white light (see Figure S8), confirming the photogeneration of O_2_(^1^Δ_g_) under polychromatic irradiation.

In addition to the photogeneration of O_2_(^1^Δ_g_), other reactive oxygen species (ROS) contributed slightly to overall photooxidation. The photoproduction of singlet oxygen via energy transfer from triplet state of Rose Bengal photosensitizer is dominant (Φ_Δ_∼0.75 in water), but the alternative channel is electron transfer to oxygen that leads to the formation of superoxide O_2_^–^, following fast disproportionation to traces of more stable H_2_O_2_, resulting in a typical postirradiation oxidation effect with slow kinetics, as observed during irradiation of sample 3-RB-NOP (Figure 6C). The photogeneration of H_2_O_2_ was confirmed by a scopoletin detection assay (Figure S9).

### 3.7 Photogeneration of NO

The ability of samples with NOP (3-NOP, 3-RB-NOP) to photogenerate NO was confirmed via the Griess assay, a commonly used colorimetric method for the detection of NO photogenerated from NOP encapsulated in various polymeric nanomaterials, as NO is easily oxidized to nitrate ions in saturated aqueous solutions in air.^45^ The Griess test is known for its lower sensitivity, as it can detect photogenerated NO down to micromolar (approximately 0.5 μM) concentrations.^46^ This drawback was partially compensated by extending the irradiation time to 30 min. As demonstrated in Figure 6E, a clear difference was observed between the irradiated and nonirradiated samples capable of generating NO (3-NOP, 3-RB-NOP) and 1 (without NOP). Notably, 3-NOP photogenerated more NO radicals than 3-RB-NOP did because of its higher absorbance at the excitation wavelength (λ_exc._ = 414 nm, Figure 4).

### 3.8 Antibacterial Activity

Figure 6E shows an example of bacterial colonies on agar plates after inoculation with *E. coli* harvested from the surfaces (irradiated or stored in the dark) of 1, 2-RB, 3-RB, and 3-RB-NOP and incubation overnight. The agar plates that were inoculated with bacteria from 1 stored in the dark and/or irradiated by light were used as negative controls. Strong photodynamic inactivation, i.e., a reduction in CFU, was observed for samples 2-RB, 3-RB, and 3-RB-NOP, which were irradiated with green light (λ_exc._ = 515 nm) for 5 and 10 min, in contrast to both controls (1 stored in the dark or irradiated). This effect is attributed to the efficient photogeneration of strong antibacterial O_2_(^1^Δ_g_).^28–31^ The reduction in CFUs (Figure 6F) is consistent with the ability to oxidize the external chemical substrate with singlet oxygen (Figure 6A–C), although the range in efficiency is different. In particular, 2-RB behaves differently. The photooxidation efficacy with respect to the absorption factor of 1–10^–A^ at the excitation wavelength is relatively low among the tested samples, which is probably due to shielding and/or self-quenching effects, but the photooxidation ability slightly increases (Figure S7). In contrast to its rather average photooxidation ability, the antibacterial effect of 2-RB is the most effective. Note that there is no direct proportionality between the amount of photogenerated antibacterial species and the total antibacterial response. The overall antibacterial effect is much more complex than the simple photooxidation process via O_2_(^1^Δ_g_) and may cover the contribution of the photogeneration of other ROS via photoreaction I (Figure S9).

2-RB, 3-RB, and 3-RB-NOP exhibited slight dark cytotoxicity toward bacteria. Surface functionalization may slightly affect the apparent contact angle (Figures 2 and S2), and therefore, surface bacterial adhesion may influence dark toxicity as well as photodynamic inactivation.

The antibacterial tests were also performed on 1, 3-NOP, and 3-RB-NOP, where pristine membrane 1 served as a control, and blue light was used for excitation of NOP for photogeneration of NO radicals. The tests revealed a less efficient but still notable antibacterial effect of NO compared to membranes with O_2_(^1^Δ_g_) photogeneration. Both the 3-NOP and 3-RB-NOP membranes exhibited comparable antibacterial effects, although 3-NOP resulted in the photogeneration of NO radicals with greater efficiency (Figure 6D). Blue light has some antibacterial effect itself^47^ complicates evaluation and masks the net effect of NO radicals. In contrast, no cytotoxic effect of green light itself was detected. No leakage of RB or NOP from the samples used was detected. The antibacterial assay also combined 5 min of green and 5 min of blue light irradiation of samples 1, 2-RB, 3-RB, 3-NOP, and 3-RB-NOP (for experimental details see Supporting Information). The results (Figure S10) demonstrate effective antibacterial activity, particularly in samples with a high surface concentration of RB (2-RB) and in those combining NOP and RB.

### 3.9 Cytocompatibility Assessment of Photosterilized Membranes

As follows from the antibacterial tests, 15 min of irradiation with green or green/blue light should be sufficient for efficient photosterilization of the sample surfaces. The cytocompatibility of photoactive nanofiber membranes sterilized via green and/or blue light irradiation was assessed for tissue engineering applications via human adipose-derived stem cells (ADSC). The evaluation focused on cell adhesion, proliferation, and morphology. Based on prior findings^48,49^ that glutaraldehyde enhances sample rigidity (by a cross-linking effect) and cell adhesion and that O_2_(^1^Δ_g_) photogeneration is more effective in terms of the antibacterial effect, testing was focused on four groups: pristine PCL (1), aminolyzed PCL (2), and two photoactive samples containing glutaraldehyde, which photogenerate O_2_(^1^Δ_g_) (3-RB) or both O_2_(^1^Δ_g_) and NO radicals (3-RB-NOP). Samples 1 and 2 were chosen primarily as controls and to evaluate the effect of aminolysis itself. Two photoactive samples, namely, 2-RB and 3-NOP, were excluded from the cytocompatibility tests. 2-RB does not contain glutaraldehyde but partially produces longer-lived H_2_O_2_, which could be toxic to ADSCs. On the basis of the antibacterial tests, photosterilization based solely on the photogeneration of NO from 3-NOP may not be sufficient.

After 8 days of cultivation, cell proliferation was slightly greater on the aminolyzed membrane 2 and significantly greater on the photoactive 3-RB and 3-RB-NOP membranes than on pristine control 1 (Figure 7A).

**Figure 7:**
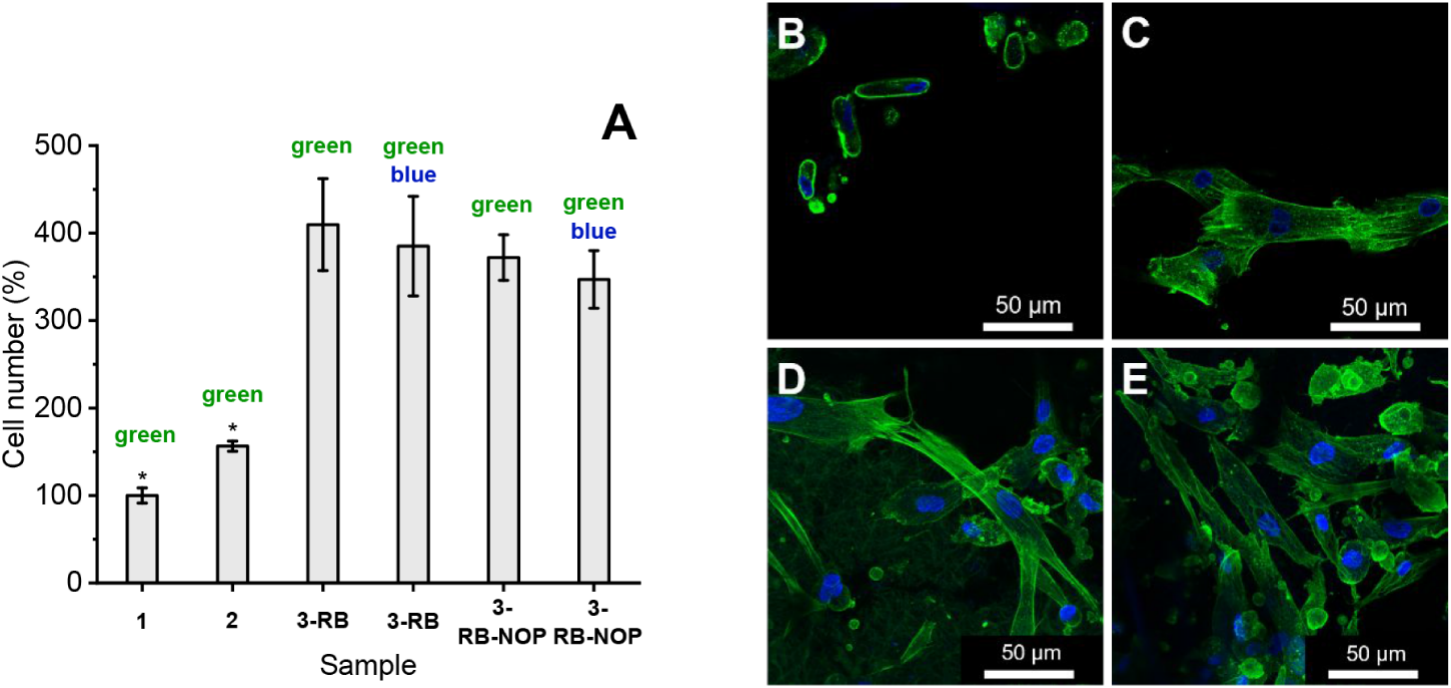
Relative ADSC numbers (with respect to control 1) found on the surface of membranes 1, 2, 3-RB, and 3-RB-NOP were detected 8 days after photosterilization (green or green and blue assigned samples after green or green and blue light photosterilization, respectively), and the number of cells seeded at each experimental point was calculated as the mean ± SD from 4–8 independent samples (40–60 microphotographs) via ANOVA and the Student–Newman–Keuls test. A statistically significant difference (*p* ≤ 0.05) in comparison with the other groups is marked above the column (*). (A). Immunofluorescence images illustrating the cell morphology and spreading of adipose stem cells (340 000 ASDC/cm^2^ on the surface of pristine membranes 1 (B), 2 (C), 3-RB (D), and 3-RB-NOP (E), 8 days after green and blue light sterilization and cell seeding, visualized by staining F-actin with TRITC (green). The cell nuclei were counterstained with DAPI (blue). The samples were photographed with a Leica Stellaris 8 confocal microscope (40× objective, zoom 2×).

These findings indicate that surface modifications and sterilization methods are nontoxic and cytocompatible, actively supporting cell growth. Note that approximately 30% of the irradiated samples 1 and 2 (nonphotoactive membranes) and all the samples without photosterilization suffer from bacterial contamination and were therefore excluded from the above-mentioned statistics. Sample 3-RB (O_2_(^1^Δ_g_) only) demonstrated slightly greater cell proliferation than sample 3-RB-NOP (O_2_(^1^Δ_g_) and NO radicals), but the differences were not significant and were roughly within a statistical error that may reflect the inhomogeneity of the samples.

Immunofluorescence analysis confirmed these findings. While the cells on the pristine PCL showed poor spreading, the cells on all the modified membranes, especially those photoactive with glutaraldehyde (3-RB and 3-RB-NOP), exhibited a well-spread morphology with a clearly organized F-actin cytoskeleton, indicating excellent cell adhesion and favorable cell–material interactions (Figure 7 B, C, D, E). For more details about the cytocompatibility assessment and other images, see Figure S11.

## 4 Conclusions

This study investigated the fabrication, functionalization, and photochemical and photobiological evaluation of aminolyzed electrospun polycaprolactone nanofiber materials. These materials were developed with and without a glutaraldehyde linker to facilitate the external covalent attachment of two distinct photoactive compounds activated by visible light: a Rose Bengal photosensitizer and a NO photodonor. The surfaces of the resulting photoactive nanofiber materials demonstrated potent antibacterial efficacy against *E. coli* upon irradiation with green and blue light. Green light irradiation induces an antibacterial effect and surface sterilization primarily through the photogeneration of singlet oxygen, with a partial contribution from hydrogen peroxide. Blue light irradiation triggers the photogeneration of nitric oxide from the conjugated NO photodonor, which also contributes to its antibacterial properties. Notably, the nanofiber membranes fabricated using the glutaraldehyde linker exhibit increased rigidity due to a cross-linking effect. Additionally, these photosterilized materials display high cytocompatibility with adipose-derived stem cells. The unique combination of visible light-controlled antibacterial and surface sterilization capabilities, coupled with excellent cytocompatibility, suggests significant potential for these nanofiber materials in a range of biomedical applications, particularly within the field of tissue engineering.

## Supplementary Material



## Notes

Experimental details. Additional characterization of the materials. Details of the photochemical and photobiological experiments (PDF)

## Abbreviations

RB: Rose Bengal
NOP: NO photodonor (4-chloro-2-(trifluoromethyl)-1-nitrobenzene)
PCL: polycaprolactone
O_2_(^1^Δ_g_): singlet oxygen
Membranes: 1, pristine; 2, after aminolysis
2-RB: after aminolysis with RB
3: after aminolysis with bonded glutaraldehyde
3-NOP: after aminolysis with bonded glutaraldehyde and NOP
3-RB: after aminolysis with bonded glutaraldehyde and RB
3-RB-NOP: after aminolysis with bonded glutaraldehyde, RB, and NOP
