# Supplementary material for: Aminolyzed Polycaprolactone Nanofiber Scaffolds with Visible Light-Activated Sterilization for Tissue Engineering Applications
Source: Biomacromolecules. 2025 Oct 7;26(11):7866–76. doi: 10.1021/acs.biomac.5c01352 (PMC12606562; doi:10.1021/acs.biomac.5c01352)
Supplement: Supplementary file 1 [file bm5c01352_si_001.pdf]

Supporting Information

Aminolyzed Polycaprolactone Nanofiber Scaffolds with Visible Light-Activated Sterilization for Tissue Engineering Applications

Robert Willimetz,[†] *Pavel Kubát,* [‡] *Jan Svoboda,* [§] *Jana Musílková*[⊥] *and Jiří Mosinger*[†*]

†Faculty of Sciences, Charles University in Prague, Hlavova 2030,

128 43 Prague 2, Czech Republic

‡J. Heyrovský Institute of Physical Chemistry, v.v.i., Academy of Sciences of the Czech Republic, Dolejškova 3, 182 23 Prague 8, Czech Republic

§Institute of Macromolecular Chemistry, Academy of Sciences of the Czech Republic, Heyrovského nám. 2, 162 00 Prague 6, Czech Republic

⊥Institute of Physiology of the Czech Academy of Sciences, Vídeňská 1083, 142 00 Prague 4, Czech Republic

Content

	Experimental details ………………………………………………	**S-2**
**Fig. S1**	SEM micrographs of nanofiber membranes samples ..………………	**S-4**
**Fig. S2**	Illustration of ACA measuring ..……………………………………..	**S-4**
**Fig. S3**	Orange II dye experiment ………………………………………….	**S-5**
**Fig. S4**	Orange II dye calibration curve ..…...…………………………….	**S-5**
**Fig. S5**	Glutaraldehyde binding prove ………….………………………..	**S-6**
**Fig. S6**	Leakage of RB and NOP …………………………………………..	**S-6**
**Fig. S7**	Photooxidation abilities of samples ………………………………..	**S-7**
**Fig. S8**	Photooxidative activity of 3-RB-NOP under white light irradiation ..	**S-7**
**Fig. S9**	Hydrogen peroxide detection (Scopoletin test) ..……………………..	**S-8**
**Fig. S10**	Antibacterial test – combined green and blue light irradiation ...……..	**S-8**
**Fig. S11**	Biocompatibility test after green light irradiation …...………….….	**S-9**
**Fig. S12**	Wavelength of used LED light sources ...……………………………..	**S-10**

Experimental details

Detection of hydrogen peroxide. A sample (2 cm^2) of photoactive nanofiber membrane containing RB (2-RB, 3-RB, 3-RB-NOP) fixed on quartz glass was immersed in a scopoletin solution (0.17 mM) in 0.01M PBS (pH = 7.4) and irradiated by Rubylux green LED light ($\lambda_{exc.}$ = 515 nm) for 30 min. After irradiation, 10 µL of HRP solution (1 mg/mL) was added. Then, the solution was incubated for 5 min at 25 °C, and fluorescence kinetics of scopoletin quenching by photogenerated H_2O_2 and catalyzed by HRP was detected at 462 nm ($\lambda_{exc.}$ = 350 nm). The results were subsequently corrected to the light/dark control (pristine PCL only).

Detection of nitric oxide (NO). A sample of membrane (2 cm^2) was fixed on a quartz glass and immersed in 2.0 mL 0.01M PBS solution in a quartz cuvette (4 cm^3) for 10 min in the dark. A 400 nm cut off filter was placed in front of the sample and each sample was irradiated for 30 min by an 18W Rubylux blue LED ($\lambda_{exc.}$ = 414 nm) from a distance of 27 cm. After the irradiation, 500 µL of freshly prepared 0.7% sulfonylamide solution in 5% phosphoric acid and 500 µL of 0.2% N-(1-naphthyl)ethylenediamine solution in 5% phosphoric acid were added to the previously irradiated

PBS solution. The cuvette was placed in the dark for 10 min, and then absorbance at 540 nm was monitored, indicating the formation of purple azo-dye. The experiment was conducted for each sample three times with and without irradiation. The results were subsequently corrected to the light/dark control (PBS only).

Antibacterial tests. A culture of *E. coli* DH5α (Invitrogen, California, USA) with the plasmid pGEM11Z (Promega, Wisconsin, USA) was incubated at 37 °C under mild shaking at 100 rpm. The incubation was finished once the value of the suspension absorbance reached approximately 1 at 600 nm. The prepared culture was diluted 350× to the desired concentration in PBS solution. In the Petri dishes, the samples 1, 2-RB, 3-RB, and 3-RB-NOP (1 cm^2 pieces) were put on cotton pads pre-moistened in sterile PBS solution with 0.01% Tween 20. Subsequently, the Petri dishes were closed, and the samples on wet cotton pads were placed in an incubator set to 37 °C. This step served to wet the samples for a period of 10 min. The surfaces of the samples were inoculated with 5 µL of the diluted bacterial suspension (approx. 1227 ± 146 CFU) and then either irradiated with Rubylux green LED ($\lambda_{exc.}$ = 515 nm) source for 5 or 10 min from a distance of 20 cm, or left in the dark for 10 min. Samples were then transferred to Eppendorf tubes containing 495 µL of sterile PBS and thoroughly shaken by IKA Vortex 3 (IKA-Werke, Germany) for 30 s. After vortexing out the bacteria, the samples were removed, and 150 µL of the suspension was transferred to sterile agar plates. Each sample was tested in triplicates. The plates were incubated for 18 hours in the dark at 37 °C. After bacterial growth, agar plates were photographed, and colonies were counted using OpenCFU software.

Analogously were treated samples 1 (control), 3-NO, and 3-RB-NO. The surfaces of the samples were again inoculated with 5 µL of the diluted bacterial suspension (approx. 325 ± 74 CFU) but this time the samples were irradiated with Rubylux blue LED ($\lambda_{exc.}$ = 414 nm) source through UV cut-off filter (400 nm) glass for 5 or 15 min for photogeneration of NO radicals or kept in the dark for 15 min. The next procedure was the same as described above.

In the final experiment, samples 1 (control), 2-RB, 3-RB, 3-NOP, and 3-RB-NOP were subjected to sequential green and blue light irradiation. Each sample was inoculated with 5 µL of the diluted bacterial suspension (approximately 106 ± 12 CFU). Irradiation was performed using a Rubylux green LED light source ($\lambda_{exc.}$ = 515 nm) for 5 min, immediately followed by Rubylux blue LED

irradiation ($\lambda_{exc.}$ = 414 nm) for an additional 5 min. Control samples were kept in the dark for 10 min. The next procedure was the same as described above.

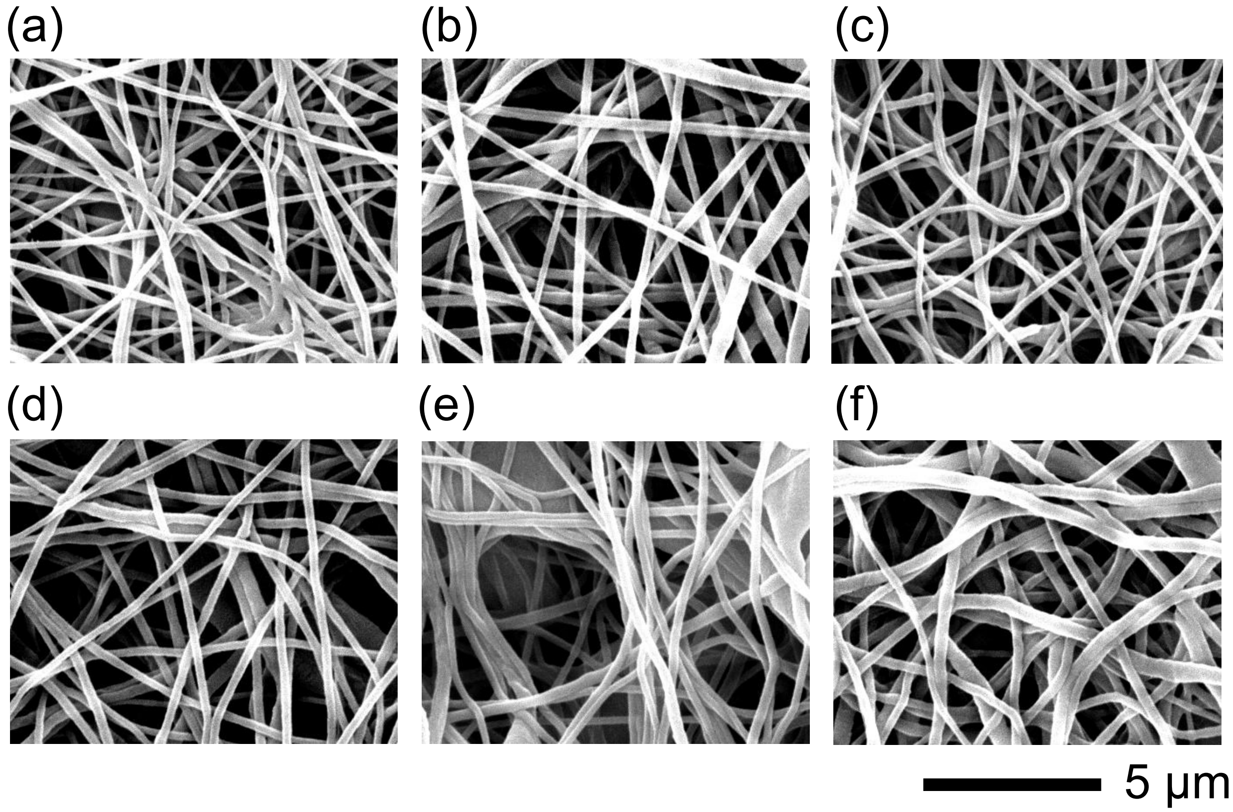

Figure S1. SEM micrographs of nanofiber membrane samples 1 (a), 2 (b), 2-RB (c), 3 (d), 3-RB (e), and 3-RB-NOP (f).

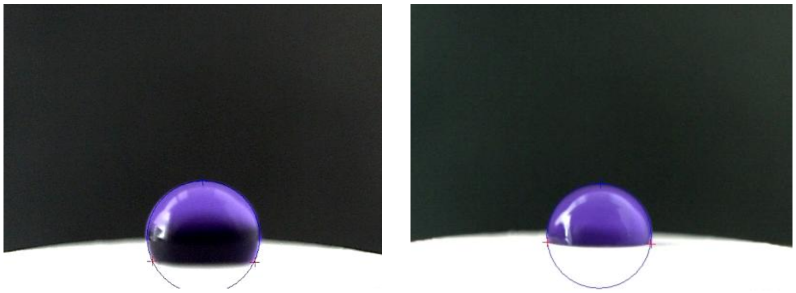

Figure S2. Illustration of ACA measuring of samples 1 (left) and 2 (right).

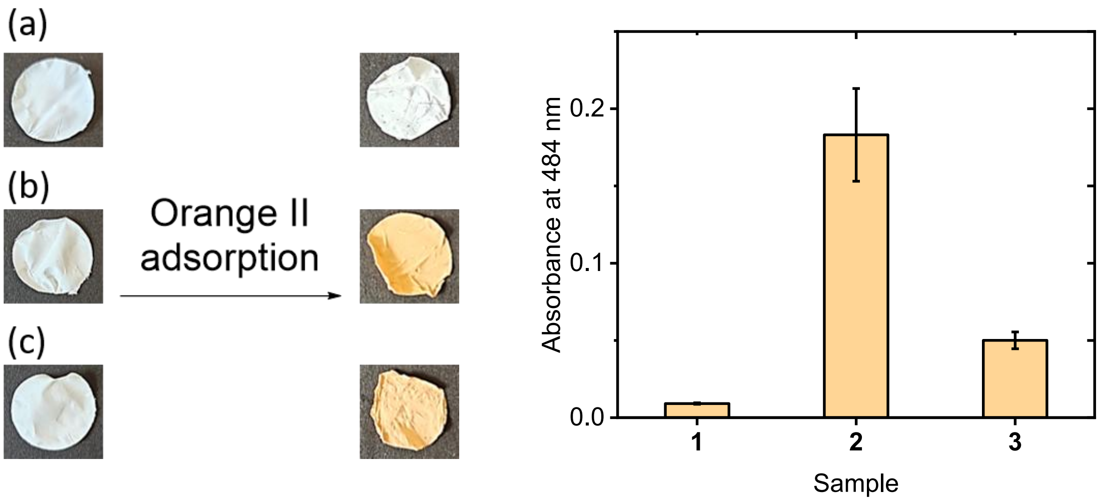

Figure S3. Images of the membrane 1 (a), 2 (b), and 3 (c) before and after adsorption of the Orange II dye (left). Change of the absorbance values at 484 nm after desorption of the Orange II dye (right) from the samples 1, 2, and 3.

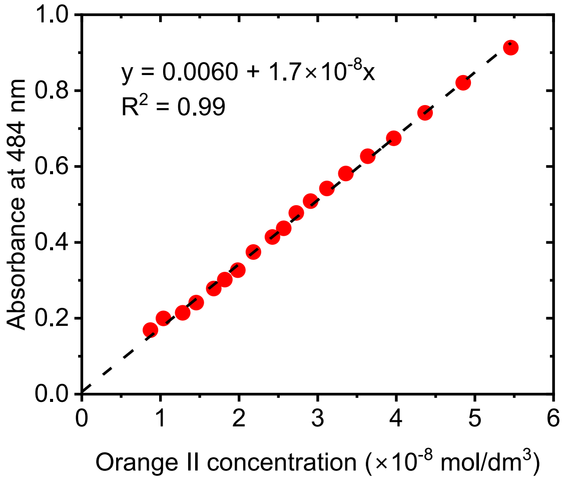

Figure S4. The linear calibration curve of Orange II dye.

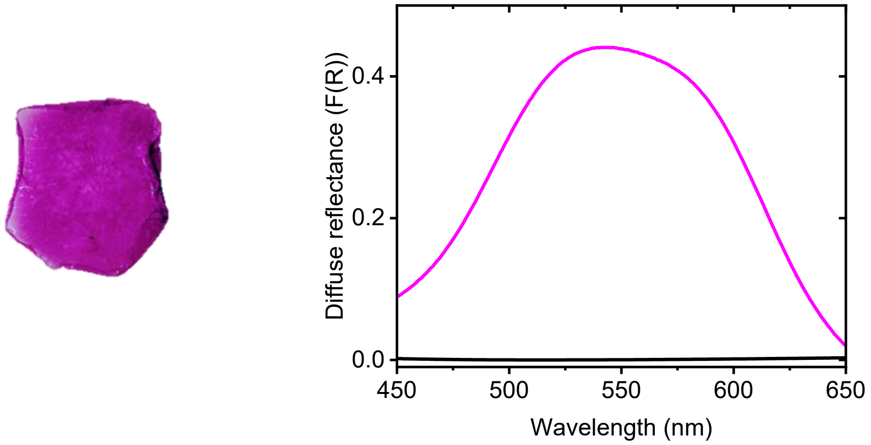

Figure S5. A photo of membrane 3 after immersion and incubation in Schiff's reagent (left) and corresponding UV-vis diffuse reflectance spectra before (black line) and after (magenta line) the addition of Schiff's reagent (right).

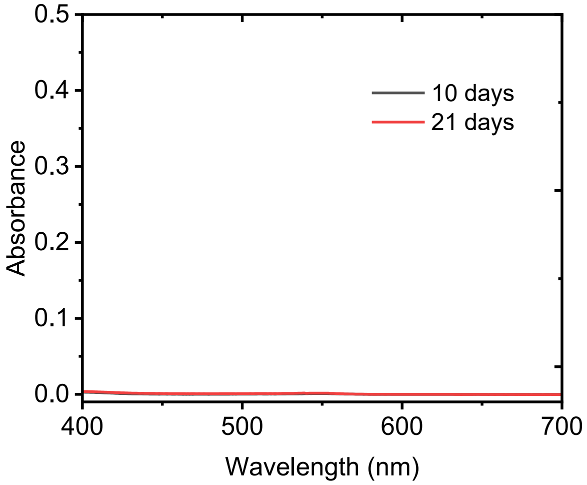

Figure S6. Absorption spectra of the 0.01M PBS washing buffer solution after 10 (black) and 21 (red) days after 3-RB-NOP immersion at 37 °C in the dark.

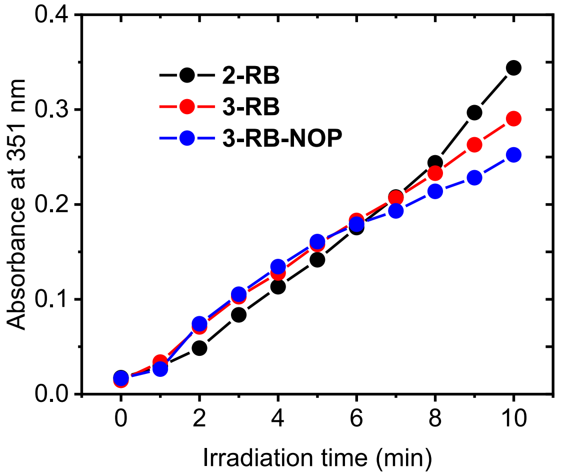

Figure S7. Dependence of the absorption of triiodide at 351 nm on the irradiation time upon irradiation of samples 2-RB, 3-RB, and 3-RB-NOP with a green LED ($\lambda_{exc.}$ = 515 nm) source.

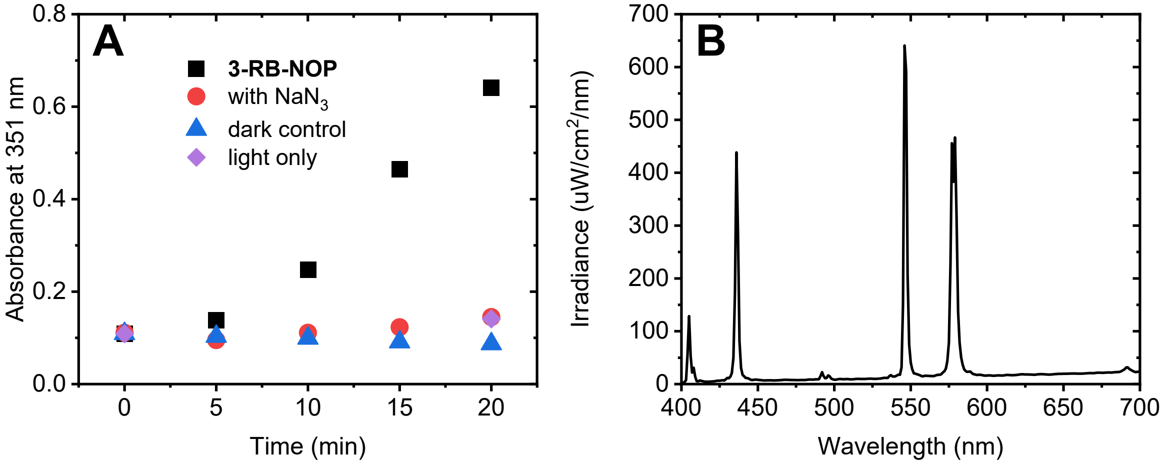

Figure S8. A: Dependence of the absorption of triiodide at 351 nm on the irradiation time upon irradiation of iodide detection solution with 3-RB-NOP after irradiation of white light (500W Xe-lamp, Newport) (black) and controls, illustrating the effect of the presence of NaN_3 quencher (red), absence of light (blue) and irradiation of the detection solution without the sample. B: Irradiance of the corresponding Xe-lamp.

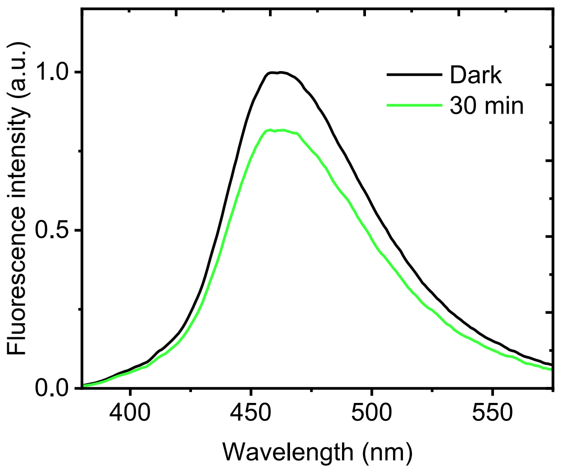

Figure S9. Scopoletin detection assay for sample 2-RB. Emission spectra of the scopoletin and HRP solution containing sample 2-RB, before (black line) and after 30 min of irradiation (green line).

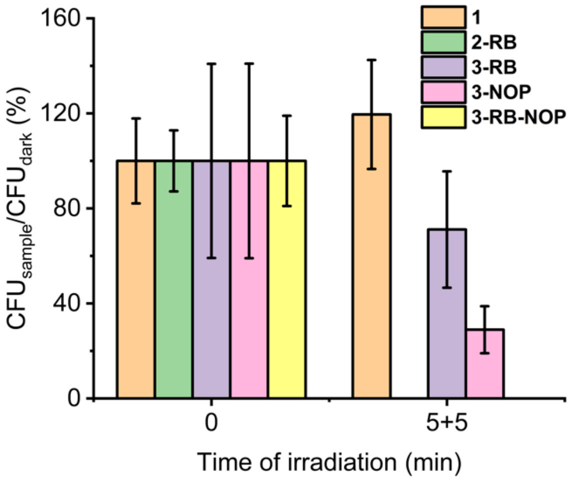

Figure S10. Antibacterial test: CFU of *E. coli* on agar plates after inoculation with bacteria harvested from the surfaces of 1, 2-RB, 3-RB, 3-NOP and 3-RB-NOP stored in the dark (0 min) and after 5 min of green light (λ_{exc}= 515 nm) and 5 min of blue light ($\lambda_{exc.}$ = 414 nm) irradiation (5+5) and incubation overnight.

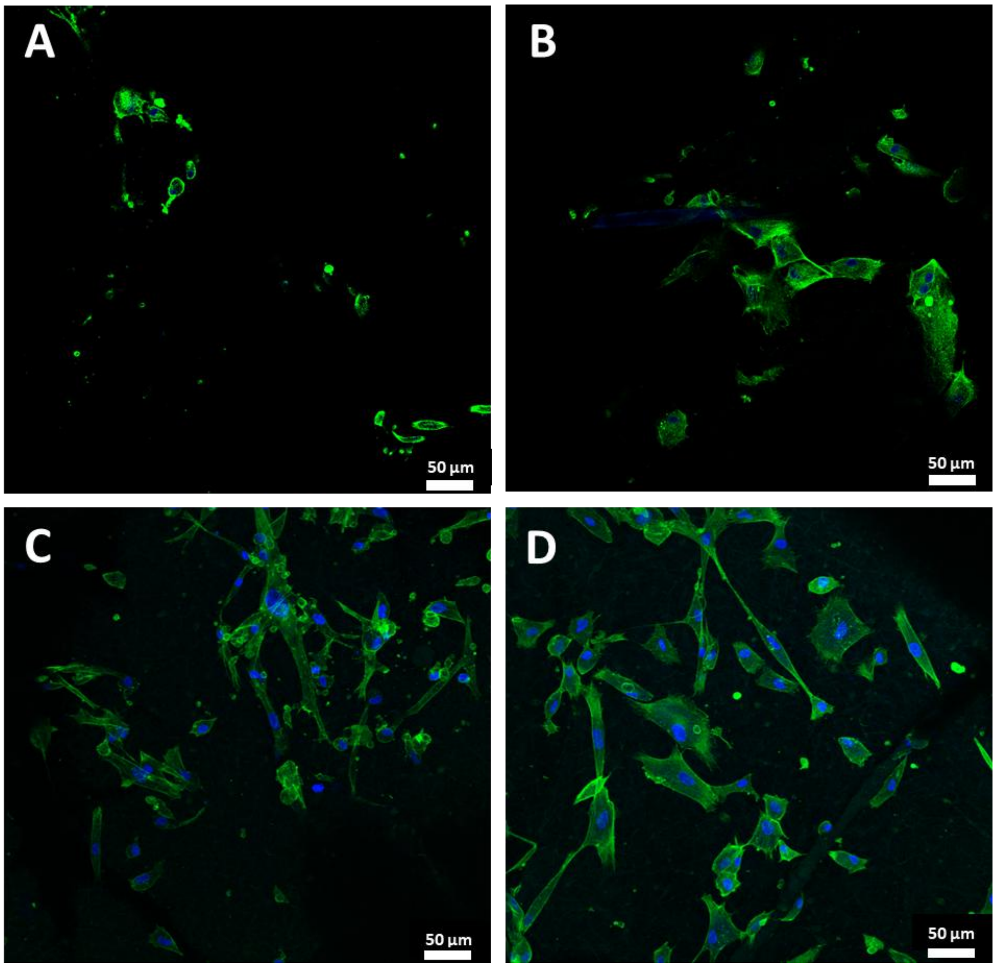

Figure S11. The spreading of adipose stem cells (30 000 ADSC/cm^2) on the surface of pristine PCL nanofiber membrane 1 (A), 2 (B), 2-RB (C), and 3-RB-NOP (D), eight days after green light sterilization and the cell seeding, visualized by staining of F-actin with TRITC (green). The cell nuclei were counterstained by DAPI (blue). The samples were photographed by Leica Stellaris 8 Confocal microscope (objective 40×).

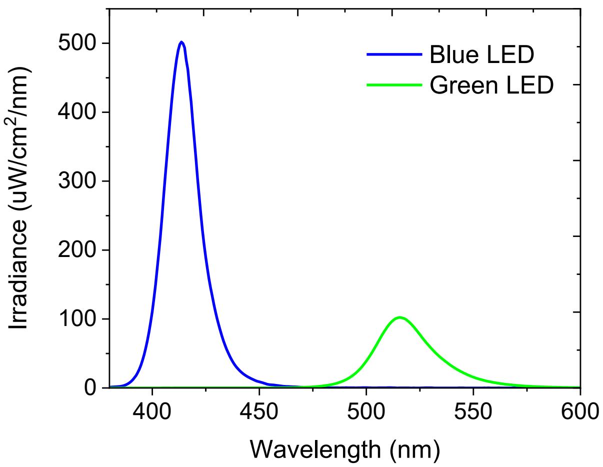

Figure S12. Wavelengths of the blue ($\lambda_{exc.}$ = 414 nm) and green ($\lambda_{exc.}$ = 515 nm) LED sources.
